# Assessing Military Professionals’ Endorsement of Decision-Making Assumptions: An Exploratory Factor Analysis

**DOI:** 10.3390/bs16040604

**Published:** 2026-04-18

**Authors:** Jostein Mattingsdal

**Affiliations:** Section for Seapower and Naval Leadership, Royal Norwegian Naval Academy, Norwegian Defense University College, 5165 Bergen, Norway; fhs.sksk@mil.no

**Keywords:** recognition-primed decision making, exploratory factor analysis, adaptive decision making, military professionals, VUCA environments

## Abstract

This study examines how military professionals interpret claims about decision making in volatile, uncertain, complex, and ambiguous (VUCA) environments. A survey of active-duty personnel (N = 225) from the Norwegian Armed Forces (2024–2025) was used to assess whether endorsement of Klein’s 11 decision-making claims reflects a unified construct or several distinct decision-making beliefs. After removing two items with insufficient communalities, exploratory factor analysis (principal axis factoring with Oblimin rotation) was conducted on the remaining nine items. Sampling properties were adequate (KMO = 0.741; Bartlett’s χ^2^ (36) = 282.86, *p* < 0.001). Comparative model testing indicated that a two-factor structure provided a better fit than a unidimensional model, accounting for 29.24% cumulative variance. The resulting dimensions—Planning/Structure (e.g., “Identify and mitigate risks,” loading 0.618) and Analytic/Evidence-based practices (e.g., “Prefer logic over intuition,” loading 0.556)—showed acceptable internal reliability (α = 0.65 and α ≈ 0.71). These findings suggest that military professionals’ endorsement of Klein’s framework is not unidimensional but instead reflects two complementary attitudes about effective decision making. This bifactorial structure offers a theoretically grounded basis for advancing research on adaptive decision making in the military and other high-stakes operational contexts.

## 1. Introduction

Military leadership in contemporary operational environments demands cognitive agility that surpasses traditional staff-planning assumptions (Olsen, 2025). Faced with volatility, uncertainty, complexity, and ambiguity (VUCA)—from asymmetric warfare to humanitarian crises—decision-makers must employ competencies that exceed the assumptions of classical rationalist models (Codreanu, 2016). Such models typically emphasize systematic generation and comparison of multiple options, structured evaluation across predefined criteria, and probabilistic reasoning. However, research from naturalistic operational settings consistently demonstrates that these analytical strategies often fail to capture how experienced practitioners make decisions under time pressure, uncertainty, and rapidly changing conditions.

Klein’s (1993, 2017) Recognition-Primed Decision (RPD) theory offers a compelling alternative to these prescriptive, universal decision rules. Derived from extensive studies of fireground commanders, military leaders, and other experts, the RPD model explains how experienced individuals frequently make rapid and effective decisions without generating or comparing multiple options. The model consists of two cognitive processes: (1) Situation assessment, in which decision-makers recognize relevant cues, form expectancies, and identify plausible goals based on prior experience. (2) Mental simulation, in which they imagine how a selected course of action will unfold in practice and evaluate whether it will succeed or require modification. This recognitional process enables action despite ambiguity and change. If the mental simulation reveals flaws, the option is modified; if the flaws cannot be resolved, the decision-maker shifts to the next most typical response—again without generating a large set of alternatives.

In his book *Streetlights and Shadows*, Klein (2009) summarizes 11 decision-making claims that describe common conceptions about how experts decide. Some align with analytical decision models (e.g., collecting all available evidence, comparing alternatives), whereas others reflect naturalistic decision-making concepts central to RPD, such as cue recognition and satisficing. Because these claims originate from different scholarly traditions, they are unlikely to form a unified belief structure. This distinction provides a clear theoretical basis for expecting multiple underlying attitudinal dimensions when practitioners assess Klein’s statements. Although Klein’s descriptions of real-world decisions are widely referenced in military training—such as Joint Doctrine Publication 04: Understanding and Decision-Making (DCDC, 2016)—their empirical structure, whether practitioners endorse them as a single coherent framework or as multiple distinct beliefs about effective decision-making practices, remains untested.

The present study addresses this gap by examining how 225 military professionals interpret and organize Klein’s 11 decision-making claims when presented as attitude statements. Importantly, the current analysis does not attempt to measure decision performance or cognitive processing. Instead, it assesses beliefs about effective decision-making practices. Rather than imposing a predefined structure, it adopts an exploratory psychometric approach to identify the latent dimensions that best represent participants’ responses. This approach allows investigation of the following research question (RQ1): Does endorsement of Klein’s decision-making claims form a single coherent dimension, or do military professionals organize them into multiple beliefs about effective decision-making practices?

### 1.1. Background

#### 1.1.1. The VUCA Imperative

Modern military operations are defined by VUCA conditions where static protocols rapidly become obsolete (Franke, 2011). Effective response requires a twofold competency: procedural rigor (e.g., structured planning, role clarity) to manage controllable elements, and adaptive reasoning (e.g., probabilistic thinking, information triage) to navigate emergent uncertainty (Smithson & Ben-Haim, 2015). This tension—between structured discipline and agile adaptation—reflects Klein’s (2017) argument that decision efficacy is determined by contextual fit rather than universal prescription.

For the Norwegian Armed Forces, these VUCA dynamics become acutely evident in two distinct operational domains: strategic competition in the High North and international counterinsurgency operations. In the Arctic, the security environment is characterized by unpredictable great-power posturing. Russia’s modernization of Cold War-era bases, deployment of advanced weapon systems, unannounced snap exercises, and simulated attacks against Nordic states have eroded stability and transparency, contributing to what Wither (2021) identifies as an emerging Arctic security dilemma in which ambiguous signaling and deteriorating dialogue increase the risk of unintended escalation. These dynamics generate pervasive uncertainty, forcing Norwegian commanders to rely on rapid cue recognition and adaptive judgment to avoid miscalculation.

Concurrently, Norway’s operational experience in Afghanistan (2007–2012) exemplifies VUCA challenges at the tactical and operational levels. Commanders confronted ambiguous threat patterns, contradictory intelligence, contested narratives, and fluid local alliances. As Laugen (2016) documents, decisions were frequently made under high cognitive load and unclear cause–effect relationships, requiring reliance on recognitional, experience-driven processes rather than purely analytical optimization.

These multifaceted realities—from high-stakes Arctic deterrence to complex expeditionary missions—collectively underscore the necessity for cognitive agility, rapid pattern recognition, and context-sensitive reasoning within the Norwegian national security framework. These real-world VUCA conditions make Klein’s model not just an interesting theory but something military leaders must rely on in practice.

#### 1.1.2. Theoretical Framework

Klein (2009) distinguishes between “streetlight” environments, where structured planning reliably supports performance, and “shadow” environments, where ambiguity, uncertainty, and time pressure undermine rule-based approaches. His 11 claims (pp. 8–10) critique common conceptions about expert judgment and highlight how trained individuals actually operate. These claims describe the following:

1. Teaching people procedures helps them make decisions and perform tasks more skillfully.
2. Decision biases distort the quality of decision-making processes.
3. Successful decision-makers rely on logic and statistics instead of intuition.
4. To make a good decision, generate several options and compare them to choose the best one.
5. We can reduce uncertainty in decision situations by collecting more information.
6. It is bad to draw early conclusions; wait to see all the evidence before making decisions.
7. To help people learn to make better decisions, give them feedback on the consequences of their actions.
8. To understand a situation, people draw inferences from available data based on their expertise.
9. The starting point for any project is to obtain a clear description of the project’s goal.
10. Our plans will succeed more often if we identify the biggest risks and then find ways to reduce them.
11. Leaders can create common ground for decision making by assigning roles and clarifying rules in advance.

Collectively, Klein’s discussion of these claims challenges traditional, normative models of decision making and instead portrays expert judgment as adaptive, pattern-based, and contextually sensitive. This aligns with broader VUCA scholarship showing that professional performance in turbulent environments requires both structural clarity and adaptive cognition (Abidi & Joshi, 2018; Elkington et al., 2017).

To show how these claims fit within real-world cognitive demands, Klein (2009) organizes expert thinking into three interdependent cognitive functions: sense making, decision making, and adapting (p. 7). These strands appear repeatedly across his studies of firefighters, military commanders, and pilots, and together form the core of his framework for understanding expert performance in the face of ambiguity and change.

Although analytically distinct, these three cognitive functions operate as a single, integrated system: sense making frames what options become salient; decisions change the environment practitioners must interpret; and adaptation updates both understanding and action. Together, they form a robust model of experienced individuals’ thoughts and actions in VUCA environments, including modern military operations.

RPD theory further explains how decision-makers shift fluidly between intuitive recognition and analytical evaluation depending on situational cues (Klein, 2017). Despite the prominence of RPD frameworks in military leader-development programs—including those at the Norwegian Defense University College (Forsdahl, 2017), no study has empirically tested whether the 11 claims described by Klein (2009) constitute a single coherent construct or several distinct competencies. Recent systematic reviews of naturalistic decision making highlight the broad influence of Klein’s work, while also noting that few scholars employ psychometric or statistical methods to verify whether decision-making studies actually measure the psychological constructs they intend to assess (Reale et al., 2023).

### 1.2. Literature Review

The VUCA framework illustrates how professionals must balance procedural structure with dynamic flexibility, a tension reflected across defense, organizational, and behavioral scholarship (Abidi & Joshi, 2018; Bakhshi & Efatmaneshnik, 2025). Klein’s (2017) The RPD model provides a key theoretical lens: experienced individuals rely on pattern recognition and mental simulation in high-stakes contexts, contrasting with systematic optimization traditions (Gigerenzer, 2016). This perspective aligns with contemporary military education emphasizing leaders who integrate data-informed analysis with procedural discipline (Sjøgren & Nilsson, 2025).

Behavioral research further reinforces this duality. Cognitive heuristics and biases significantly shape judgments in constrained settings (Olguín Álvarez, 2025), yet purely bias-centric frameworks insufficiently capture the intuitive expertise observed in operational settings (N. D. Shortland et al., 2019). Leadership scholarship emphasizes that effective decision-makers require self-regulated sense making, resilience, and systems awareness to navigate ambiguity, competencies that draw simultaneously on structured protocols (e.g., risk identification, goal clarity) and adaptive reasoning (e.g., information triage, situational inference) (Elkington et al., 2017; Lues et al., 2020; Mattingsdal, 2025).

Military-specific studies similarly highlight the requirement for both planning discipline and adaptive responsiveness. Logisticians and field commanders must develop competencies for structured preparation alongside agility to respond to rapidly shifting conditions (Paparone & Topic, 2011). Parallel findings from decentralized leadership research show that empowering junior officers enhances organizational adaptability in environments where rigid hierarchical control becomes counterproductive (Boone, 2021; LeBoeuf & Doty, 2021). Collectively, these findings align directly with the factorial structure examined in the present study.

Methodologically, exploratory factor analysis (EFA) provides a rigorous approach for determining whether decision-making constructs reflect a single underlying dimension or a multifactorial structure. When latent constructs are theoretically expected to correlate—as is the case with Klein’s mutually reinforcing “planning” and “analytic” competencies—principal axis factoring with an oblique rotation is the preferred technique (Conway & Huffcutt, 2003; Sass & Schmitt, 2010). Although diverse analytical frameworks have been applied across disciplines, including Bayesian classifier models for complex pattern detection (Liu et al., 2007) and fuzzy-logic systems for representing uncertainty in poorly specified decision environments (Liberatore, 2008), statistical analyses remain indispensable for operationalizing latent beliefs about effective decision-making practices in organizational settings such as the armed forces (Estrada et al., 2024). While business research frequently employs factor-analytic and psychometric methods to examine decision styles—such as studies using the General Decision-Making Style Inventory in managerial samples (Curşeu & Schruijer, 2012) and healthcare decision making is extensively investigated through empirical performance-based designs (e.g., clinical triage studies; Saban and Dubovi (2025)), comparable psychometric examinations within military populations remain notably limited.

Collectively, the literature converges on a central thesis: effective military decision making depends on the interplay between pre-established plans and on-the-go adjustments. This review therefore positions the present factor-analytic study as a direct empirical exploration of that dynamic within the RPD framework.

### 1.3. This Study’s Contribution

This study addresses an empirical gap by conducting an EFA to examine how Norwegian military professionals endorse the 11 decision-making claims of Klein (2009). The analysis will compare a unidimensional structure with a theoretically derived multi-factor solution to determine whether endorsement is best represented by a general belief about effective decision making or by several dimensions.

## 2. Materials and Methods

### 2.1. Participants

A total of 225 participants took part in the study. Participants ranged in age from 20 to 44 years, with a mean age of 25.90 years (SD = 4.74). The sample consisted of 177 men (78.7%) and 48 women (21.3%). Participants were recruited through convenience sampling among active-duty military professionals attending courses at the Norwegian Defense University College between winter 2024 and spring 2025. Participation was voluntary, anonymous, and uncompensated. All participants provided complete data and were included in the analyses. Initial correlation tests showed that age and sex had no significant associations with the other study variables.

### 2.2. Materials

#### 2.2.1. Instrument

The instrument consisted of an 11-item questionnaire (Q1–Q11) drawn directly from the formulations of Klein (2009) presented in Section 1.1.2. Each item represented one of the claims and was translated into Norwegian. The items therefore retain differences in grammatical form and referent focus (e.g., ‘leaders,’ ‘we,’ or general advice). This heterogeneity reflects the structure of the original claims, which were never designed as a psychometric scale, and the present instrument was intentionally constructed to preserve their meaning rather than standardize their targets.

Participants rated their agreement with each statement on a 5-point Likert scale (1 = Strongly agree, 5 = Strongly disagree). As the items are phrased as evaluative statements, the instrument is designed to capture respondents’ attitudinal endorsement of the decision-making claims, rather than actual decision-making ability or cognitive processes. The latent variables examined therefore represent belief structures, not performance constructs.

#### 2.2.2. Equipment and Software

The questionnaire was administered digitally through Microsoft (MS) Forms a web-based MS 365 application (Microsoft Corporation, Redmond, WA, USA; https://forms.microsoft.com/, accessed on 2 February 2026), hosted on the Norwegian Armed Forces’ secure network, ensuring encrypted storage and restricted access. All data analysis was conducted using IBM SPSS Statistics Version 27 (IBM Corp., Armonk, NY, USA).

### 2.3. Procedure

#### 2.3.1. Study Design

This study employed a cross-sectional correlational design aimed at identifying the latent structure of decision-making beliefs derived from the RPD framework.

#### 2.3.2. Data Collection Steps

A secure MS Forms link was distributed via the Norwegian Armed Forces internal network. Participants reviewed the study information and provided informed consent before completing demographic items (Age and Sex) followed by the questionnaire (Q1–Q11). They were explicitly instructed to evaluate how, based on their own military experience, each of Klein’s claims contributes to effective decision making in operational contexts. All responses were automatically stored in encrypted form on the secure network. No intervention or experimental manipulation was used. The average time to complete the study was 10 min and 30 s.

### 2.4. Ethical and Institutional Approval

This study received formal approval from the Norwegian Armed Forces Research Board and was registered in the Norwegian Research Information Repository (NVA) under Project-ID 2723803. All procedures adhered to recognized Norwegian research-ethics norms as outlined by the National Committee for Research Ethics in the Social Sciences and the Humanities (NESH, 2024).

### 2.5. Data Analysis

Data were analyzed using EFA following standard psychometric procedures (Watkins, 2018):

1. Kaiser–Meyer–Olkin (KMO) measure and Bartlett’s Test of Sphericity were used to confirm factorability.
2. Principal axis factoring (PAF) served as the extraction method.
3. Direct Oblimin rotation allowed for correlated factors, consistent with theoretical expectations that cognitive processes in complex decision environments interact.
4. Communalities, inter-item correlations, and the pattern matrix were examined to identify weak or conceptually misaligned items.
5. Eigenvalues and scree plot inspection guided the final determination of factor number.

### 2.6. Methodological Considerations

Klein’s 11 decision-making claims originate from conceptually heterogeneous traditions, including prescriptive analytic models, naturalistic decision making, heuristics, and sociopolitical perspectives (Balogun et al., 2008). Because these traditions lack a shared theoretical foundation, there is no empirical basis for assuming that the claims form a single latent construct. Moreover, decision-making scholarship spans multiple competing paradigms—such as expected utility theory, prospect theory, risk–value models, heuristic frameworks, and process-oriented models (Fox et al., 2015)—further underscoring the need for an exploratory approach. For these reasons, EFA is the most appropriate method for identifying how respondents organize their attitudinal endorsement of the claims reviewed by Klein (2009).

It is important to note that EFA is designed to uncover potential latent structures rather than to confirm predetermined measurement models. Consequently, EFA cannot provide the full range of validity evidence required in later stages of scale development, including confirmatory factor analysis, convergent and discriminant validity testing, or external validation in independent samples (Flora & Flake, 2017). The present study therefore positions EFA as an initial structural investigation, with subsequent validation work required to establish a more comprehensive evidence base.

## 3. Results

### 3.1. Item-Level Descriptive Statistics

Descriptive statistics indicated adequate variability across all items, full use of the response scale, and no substantial floor or ceiling effects (Table 1).

### 3.2. Assessment of Factorability

Sampling adequacy was confirmed by KMO = 0.741, which exceeds the commonly accepted minimum threshold of 0.50 for factorability and falls within the “0.70–0.79 = good” classification. In addition Bartlett’s Test of Sphericity was significant, χ^2^ (36) = 282.860, *p* < 0.001, surpassing the required *p* < 0.05 criterion and indicating that the dataset is appropriate for factor analysis (Hadi et al., 2016).

**Figure 1:**
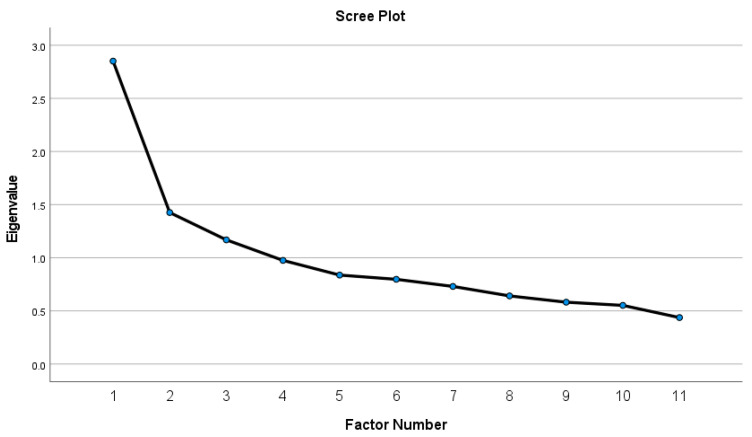
Scree plot for the initial 11-item analysis.

Initial extraction produced three eigenvalues greater than 1 (2.61, 1.41, 1.09), consistent with the Kaiser criterion for preliminary factor retention (Braeken & van Assen, 2017). However, as recommended in best-practice guidelines, inspection of the scree plot revealed a clear inflection after the second factor (see Figure 1), indicating that only the first two factors represented substantial common variance and supporting a two-factor solution (Howard, 2016).

Following initial screening, two items (Q2 and Q7) were excluded due to critically low communalities (see Table 2), indicating insufficient shared variance with the emerging factor structure. A communality of <0.30 is a commonly used threshold for identifying items that do not fit well with other variables in an EFA (Hadi et al., 2016). Examination of the initial pattern matrix further showed that these items displayed unstable or negligible loadings, supporting their removal. Although Q1 and Q5 also showed low initial communalities, their pattern-matrix loadings suggested potential substantive contributions to one of the emerging factors. Because communalities alone should not be used as the sole basis for item removal, and best-practice guidance emphasizes evaluating multiple indicators, including loadings, cross-loadings, and structural consistency, these items were retained tentatively for further evaluation (Braeken & van Assen, 2017; Howard, 2016).

Following the removal of Q2 and Q7, we re-examined the inter-item correlations among the remaining items to assess their coherence within the emerging factor structure. As shown in Table 3, most items displayed low-to-moderate positive correlations, indicating an adequate level of shared variance for exploratory factor analysis. In contrast, Q1 consistently demonstrated the weakest associations across the matrix, with several correlations approaching zero or negative. This pattern suggested that Q1 was poorly integrated with the other items and did not meaningfully contribute to the developing factor structure. Q5, however, did not show this pattern of isolation; despite some low correlations, it exhibited several coherent associations with conceptually related items. Because item-retention decisions should not rely on a single criterion and require examination of multiple indicators, Q1 was flagged as a candidate for removal, whereas Q5 was retained provisionally for further evaluation during factor extraction (Schreiber, 2021).

### 3.3. Factor Extraction

Although the initial extraction suggested a three-factor structure, the scree plot indicated that only two factors were substantively meaningful. Moreover, the third factor was defined by a single item (Q1), rendering the three-factor solution psychometrically uninterpretable (Hadi et al., 2016). Consequently, a more parsimonious two-factor model was examined and retained. In the initial two-factor extraction, Q1 loaded only weakly on Factor 1; combined with its low communality, weak inter-item correlations, and negative effect on internal consistency, the item did not meaningfully contribute to the underlying construct. Consistent with recommended criteria for factor retention (i.e., primary loadings ≥ 0.40), Q1 was removed from the final model (Howard, 2016). The remaining eight items demonstrated a clear and interpretable two-factor pattern, as shown in Table 4.

### 3.4. Factor Variance

The forced two-factor solution accounted for 29.24% of cumulative variance, with Factor 1 contributing 21.63% and Factor 2 contributing 7.60%. While modest, this explanatory power aligns with established psychometric expectations for brief attitudinal instruments (Hendrick et al., 2013). The two factors were moderately correlated (see Table 5), a pattern that is fully consistent with the use of an oblique rotation, given that factors in applied research are rarely independent and are often expected to share variance (Schreiber, 2021). This supports interpreting the two dimensions as related yet conceptually distinct beliefs about effective decision-making practices.

### 3.5. Factor Structure and Reliability

The pattern matrix loadings revealed two theoretically coherent dimensions:

- Factor 1: Planning/Structure. This factor comprised Items Q10 (0.618), Q8 (0.583), Q9 (0.523), and Q11 (0.497), reflecting systematic preparation, clear goal-setting, and risk-management processes. Internal consistency for this four-item factor was borderline acceptable (α = 0.65), with corrected item–total correlations (CITCs) ranging from 0.305 to 0.571, indicating that all retained items contributed meaningfully to the factor (Green & Yang, 2009).
- Factor 2: Analytic/Evidence-Based. This factor included Items Q3 (0.556), Q5 (0.525), Q6 (0.487), and Q4 (0.419), capturing data-driven reasoning, information search, generation and comparison of alternatives, and avoidance of premature conclusions. Reliability was robust for a short subscale (α ≈ 0.71), with CITCs ranging from 0.334 to 0.399 and stability confirmed through alpha-deletion diagnostics (Cronk, 2016).

Item retention followed established best-practice criteria for EFA. First, items were required to demonstrate substantial primary loadings (≥0.40), consistent with recommended thresholds for salient factor loadings used in applied EFA research (e.g., ≥0.37–0.40 depending on sample size; Dabbagh et al. (2023)). In addition to loading magnitude, we evaluated each item’s conceptual alignment with the emerging factor structure, reflecting guidance that item retention must consider both statistical and theoretical coherence (Schreiber, 2021). Internal-consistency criteria were also applied: specifically, we examined whether removing an item inflated or weakened overall reliability, consistent with recommended procedures for assessing internal structure in EFA-based scale development (Dabbagh et al., 2023). Finally, structural validity was supported by patterns of convergent inter-item correlations within each retained factor (e.g., Q8–Q10 = 0.42; Q5–Q6 = 0.33), in line with the expectation that items intended to measure the same construct should exhibit meaningful shared variance (Schreiber, 2021).

## 4. Discussion

This study examined whether Norwegian military professionals’ endorsement of Klein’s (2009) decision-making claims is best represented by one general factor or by multiple distinct but related beliefs about effective decision making. The findings indicate that a two-factor model, Planning/Structure and Analytic/Evidence-Based Reasoning, offers a substantially better representation of the data than a unidimensional structure. This bifactorial pattern aligns with RQ1, demonstrating that military respondents meaningfully differentiate between structured preparatory practices and analytic reasoning strategies when making decisions in operational contexts, consistent with prior work showing how experts shift between different forms of reasoning depending on the operational demands they face (Schraagen & van de Ven, 2011).

### 4.1. Interpretation of Findings

The results indicate that endorsement of the decision-making claims discussed by Klein (2009) is not homogeneous. Instead of forming a single unified belief structure, participants’ responses clustered into two theoretically coherent and interpretable dimensions:

1. Planning/Structure, comprising goal clarification, risk identification, creation of shared understanding, and systematic preparation (Q8–Q11).
2. Analytic/Evidence-Based Reasoning, comprising information gathering, logic-based evaluation, comparison of alternatives, and avoidance of premature closure (Q3–Q6).

Our outcome mirrors Klein’s (2017) assertion that decision-making competence is shaped by contextual demands, not by a universal method. In moderately ordered or “streetlight” environments, structured planning is effective; in ambiguous or rapidly changing “shadow” contexts, analytic evaluation and adaptive evidence use become more important. The two-factor structure therefore operationalizes Klein’s argument at the psychometric level: effective decision making reflects the interaction of multiple competencies rather than a single general decision orientation.

Moreover, the moderate correlation between the two factors (r ≈ 0.35) further supports this interpretation, indicating that while beliefs about effective decision making are distinct, they often co-occur, a pattern consistent with Klein’s (2017) description of skilled decision-makers shifting between complementary “gears” depending on situational cues.

Lastly, when reversed to match the scale direction used in the present study, the mean endorsement levels reported by Klein (2009, p. 9)—ranging from approximately 2.0 to 2.8 on a 5-point Likert scale—are closely matched and, in several instances, exceeded by the stronger agreement observed in the current exclusively military sample. Klein’s dataset drew from a mixed cohort of business students, armed forces personnel, and process managers, yet still reflected uniformly high agreement, suggesting these claims resonate across varied professional groups. The even stronger endorsement within our military sample—particularly for items emphasizing structured procedures, goal clarity, and risk identification, consistent with the institutionalized emphasis on planning and preparation within military doctrine (Sjøgren, 2022).

### 4.2. Alignment with Previous Research

The bifactorial structure shows strong convergence with prior empirical and theoretical work across operational psychology (Eid et al., 2025), naturalistic decision making (Zsambok & Klein, 1997), and VUCA leadership (Bennett & Lemoine, 2014).

First, military research consistently identifies a dual demand for:

- procedural rigor in controllable elements of planning (Paparone & Topic, 2011) and multinational mission command systems (Sjøgren & Nilsson, 2025).
- adaptive reasoning under uncertainty, where time pressure, ambiguity, and information scarcity dominate (N. Shortland et al., 2020; Smithson & Ben-Haim, 2015), a theme also emphasized in VUCA-oriented leadership frameworks (Abidi & Joshi, 2018; Elkington et al., 2017).

The Planning/Structure dimension parallels the structured discipline emphasized in doctrine such as Joint Doctrine Publication 04 (DCDC, 2016), which views understanding and coordination as central to effective leadership. Conversely, the Analytic/Evidence-Based dimension aligns with cognitive requirements of high-risk settings described in studies of crisis decision making (Reale et al., 2023), disaster management (Chalaris, 2023) and close-quarter combat (Berger et al., 2023), which highlight information triage, flexible reasoning, and resistance to cognitive load.

Second, the findings echo RPD theory (Klein, 1993), which posits that experts alternate between recognition-driven action and analytic evaluation when a situation is unfamiliar or uncertain. The two factors extracted in this study map closely onto these modes of functioning.

Third, the results align with research on heuristics and biases. While claims about bias (“decision biases distort our thinking”) are prominent in academic and military discourse (Olguín Álvarez, 2025), our findings—showing Q2 as the weakest statistical contributor—reinforce critiques that bias-centric models often misrepresent how real-world experts actually make decisions (Gigerenzer, 2016). This supports Watson’s (2021) argument that reductive explanatory narratives, particularly those that overemphasize cognitive limitations, tend to oversimplify practitioner expertise and misrepresent how experts actually reason and perform in real-world contexts.

Finally, the emergence of two latent factors is consistent with prior psychometric research showing that decision-making inventories often yield multiple interrelated subcomponents (Curşeu & Schruijer, 2012; Sela & Berger, 2012). This study extends that tradition into a military context, addressing an empirical gap noted in reviews of decision-making research (Reale et al., 2023) and in VUCA-oriented leadership studies that emphasize the interplay between bureaucratic rigidity and flexibility (Lues et al., 2020; Meerits & Kivipõld, 2020).

### 4.3. Item Exclusion and Conceptual Boundary Clarification

Klein’s (2009) tripartite distinction between sense making, decision making, and adapting can provide an explanation for why Q1, Q2, and Q7 failed to load on either of our two emergent factors. Q1 (procedural training) and Q7 (feedback for learning) fall outside RPD’s operational strands entirely; they reflect general learning mechanisms rather than the real-time cognitive functions that guide action under uncertainty. Their exclusion therefore suggests that military professionals do not treat pedagogical beliefs as part of the same latent structure that governs operational decision making.

Q2 (bias distortion) similarly occupies a conceptual space distinct from the RPD model. Klein (2017) argues that bias-centric narratives originate in laboratory traditions and do not meaningfully describe how experts make sense, decide, or adapt in operational environments. The item’s low communality reflects this mismatch: respondents endorsed the statement in isolation but did not associate it with either structural planning or analytic adaptation.

Taken together, these exclusions reinforce Klein’s argument that effective action in naturalistic settings depends on the interaction of sense making, decision making, and adapting—not on general learning assumptions or abstract cognitive-bias frameworks. The results thus clarify the conceptual boundaries of this study’s two-factor model and answer RQ1 by showing that only claims tied to the functional strands of RPD—namely the recognitional routines of cue detection, expectancy generation, goal identification, and typical-action selection—cohere into interpretable dimensions in the current military context (Ross et al., 2005).

### 4.4. Implications of Restricted Variance for Items

Several items showed mean scores in the 1–2 range (1 = strongly agree) with correspondingly small standard deviations (Table 1), indicating limited variance. Such compression attenuates item–item correlations, lowers factor loadings, and reduces explained variance—effects that are especially pronounced in brief attitudinal scales and well-documented in simulation studies showing that range restriction systematically underestimates factor loadings, reliability, and covariance structure recovery (Franco-Martínez et al., 2023). This restricted variance may partly reflect shared training, doctrine, and evaluative language within a relatively homogeneous military cohort.

In contrast, other items originated from more pedagogical, metacognitive, or abstract learning formulations (e.g., Q1, Q2, Q7). While conceptually relevant, these items lie further from the immediate operational decision strands reflected in the two emergent factors. As such, they aligned less strongly with the underlying latent belief structures, which helps explain their weaker factor loadings and their exclusion during the item-screening process.

### 4.5. Expertise, Age, and Measurement Considerations

Although Klein’s framework distinguishes between expert and novice decision-makers, the present study did not observe age-related differences in endorsement of the two factors. This null finding should be interpreted cautiously, as chronological age is an imprecise proxy for domain-specific expertise. Expertise develops through accumulated knowledge and deliberate practice rather than age itself (Charness et al., 2008). Moreover, self-assessed expertise shows no reliable correlation with age, with substantial individual variability observed across the adult lifespan (Van Der Heijden, 2001). Consequently, our result likely reflects the limited utility of age as a surrogate for operational experience, a limitation compounded by the unavailability of more direct indicators—such as rank or years of service—in the present dataset. The absence of these variables restricts a more nuanced examination of how military experience may shape patterns of decision-making beliefs.

### 4.6. Practical Implications

Our two-factor model has several applied implications:

First, leader development programs can purposefully isolate beliefs about effective decision making associated with structured planning (e.g., goal clarification, risk mapping, establishing common ground) from those associated with analytic reasoning (e.g., comparing options, managing uncertainty). This separation enables more precise training objectives and clearer instructional design.

Second, the two subscales provide a foundation for diagnostic self-assessment and instructor feedback during professional military education. By distinguishing planning shortcomings from analytic-reasoning deficiencies, instructors can target leadership development interventions more effectively.

Third, curricula that overemphasize structural discipline may inadvertently underprepare leaders for navigating ambiguous, time-pressured contexts. The findings therefore support a more deliberate curriculum balance between structured order-based leadership and analytic adaptability in the face of change.

Finally, After-Action Reviews (AARs) can leverage the two-factor lens to categorize decision failures and successes more precisely, thereby strengthening organizational learning and enhancing lesson-learned processes.

### 4.7. Limitations and Future Directions

While this study provides initial insights into the structure of military professionals’ decision-making beliefs, several limitations warrant consideration and guide productive avenues for future research.

A primary constraint stems from the nature of the items themselves. Klein’s 11 claims were conceived as conceptual critiques rather than as psychometric indicators. This inherent conceptual heterogeneity likely contributed to the modest variance explained by the factor solution and necessitated the removal of three items during analysis. Future work should develop and validate purpose-built measurement instruments explicitly grounded in the core constructs of naturalistic decision making.

Methodologically, this study captures attitudinal endorsement but does not assess actual decision-making behavior or the cognitive processes—such as recognition-primed or analytic reasoning—that underlie these beliefs. Testing Klein’s framework in operational settings will require methodologies capable of capturing real-time processes under contextual constraints like time pressure. Furthermore, reliance on self-reported perceptions, rather than observed behavior, suggests that future research would benefit from triangulating self-report data with behavioral measures, scenario-based assessments, or process-tracing techniques to strengthen construct validity.

The generalizability of the findings is bounded by the exclusively Norwegian military sample. The organizational culture and doctrinal emphasis of this specific force may influence how Klein’s claims are interpreted. Cross-national and cross-service replication studies are therefore essential to establish the robustness and cultural invariance of the identified factor structure.

This study represents an initial exploratory step. A comprehensive validation program should pursue several lines of evidence, including confirmatory factor analysis in independent samples, as well as examinations of convergent, discriminant, and criterion-related validity. Additionally, the cross-sectional design cannot illuminate how decision-making beliefs evolve with experience, training, or operational exposure. Longitudinal research, incorporating direct indicators of expertise (e.g., rank, mission history) and behavioral decision tasks, is needed to trace the development of planning-oriented and analytic reasoning competencies across a leader’s career.

Finally, internal consistency was assessed using Cronbach’s alpha, as the software employed did not compute McDonald’s omega. Subsequent validation efforts should utilize software capable of providing this more comprehensive reliability estimate.

Despite these limitations, this research establishes a foundational empirical understanding of how military professionals cognitively organize key decision-making beliefs within VUCA environments. It offers an empirical baseline and a clear methodological roadmap for future, more process-oriented research in naturalistic decision science.

## 5. Conclusions

This study provides the first psychometric examination of military professionals’ endorsement of the 11 decision-making claims discussed by Klein (2009). Using exploratory factor analysis on responses from 225 Norwegian Armed Forces personnel, we found that these claims do not form a single general dimension. Instead, they cluster into two distinct but related beliefs about effective decision-making practices: Planning/Structure and Analytic/Evidence-Based Reasoning. This bifactorial structure aligns with central tenets of naturalistic decision-making theory, which emphasize the interplay between structured preparation and adaptive reasoning in real-world contexts. The current study’s two-factor model also echoes the logic of mission command, which balances clear, structured guidance with adaptive, intent-based decentralized decision making. This suggests that the cognitive patterns underlying practitioners’ decision beliefs are consistent with established leadership principles in modern military doctrine.

The two-factor model offers a foundation for future research on decision-making competence in operational settings characterized by high risk, time constraints, and ambiguous information. It also provides practical value for professional military education, enabling more targeted assessment and development of leaders’ planning and analytic abilities. While the findings are limited by the single-nation sample and reliance on self-report data, they represent a step toward establishing an empirically grounded framework for studying adaptive decision making in VUCA environments. Future work should include confirmatory factor analysis, cross-cultural replication, and measures to extend and refine the model.

## Figures and Tables

**Table 1:** Descriptive statistics, SD = Std. Deviation, 1 = strongly agree; 5 = strongly disagree.

Item		Min	Max	Mean	SD
Q1	Teaching people procedures helps them make decisions and perform tasks more skillfully	1	5	1.80	0.71
Q2	Decision biases distort the quality of decision-making processes	1	5	2.16	0.85
Q3	Successful decision-makers rely on logic and statistics instead of intuition	1	5	3.00	0.86
Q4	To make a good decision, generate several options and compare them to choose the best one	1	5	1.88	0.73
Q5	We can reduce uncertainty in decision situations by collecting more information	1	5	1.83	0.76
Q6	It is bad to draw early conclusions; wait to see all the evidence before making decisions	1	5	3.04	0.88
Q7	To help people learn to make better decisions, give them feedback on the consequences of their actions	1	4	1.93	0.80
Q8	To understand a situation, people draw inferences from available data based on their expertise	1	5	1.83	0.63
Q9	The starting point for any project is to obtain a clear description of the project’s goal	1	5	1.86	0.80
Q10	Our plans will succeed more often if we identify the biggest risks and find ways to eliminate them	1	5	1.74	0.66
Q11	Leaders can create common ground for decision making by assigning roles and clarifying rules in advance	1	5	1.64	0.67

**Table 2:** Communalities, extraction method; principal axis factoring.

Item	Q1	Q2	Q3	Q4	Q5	Q6	Q7	Q8	Q9	Q10	Q11
Initial	0.18	0.08	0.21	0.25	0.17	0.24	0.21	0.28	0.20	0.37	0.18
Extraction	0.17	0.07	0.35	0.30	0.17	0.26	0.17	0.38	0.25	0.52	0.24

**Table 3:** Correlation matrix.

	Q1	Q3	Q4	Q5	Q6	Q8	Q9	Q10	Q11
Q1									
Q3	0.25								
Q4	0.26	0.23							
Q5	0.10	0.24	0.20						
Q6	0.09	0.30	0.24	0.33					
Q8	−0.07	0.06	0.15	0.08	0.19				
Q9	0.13	0.11	0.16	−0.01	0.18	0.28			
Q10	0.19	0.20	0.39	0.17	0.31	0.42	0.36		
Q11	0.15	0.06	0.16	−0.02	0.14	0.28	0.28	0.30	

**Table 4:** Pattern matrix.

Item	Factor 1 Loading	Factor 2 Loading
Q10	0.618	
Q8	0.583	
Q9	0.523	
Q11	0.497	
Q3		0.556
Q5		0.525
Q6		0.487
Q4		0.419
Q1		0.333 ^a^

Note. Extraction method: principal axis factoring. Rotation method: direct oblmin with Kaiser normalization. ^a^ = Loading below the retention threshold (≥0.40).

**Table 5:** Factor correlation matrix.

Factor	1
1	
2	0.35

Note. Extraction method: principal axis factoring. Rotation method: direct Oblimin with Kaiser normalization.

## Data Availability

All data and research materials are accessible to experts in the field upon obtaining explicit permission from the Norwegian Defense University College by reaching out to the author.

## Abbreviations

The following abbreviations are used in this manuscript:

AAR	After-Action Review
CITC	Corrected Item–Total Correlation
EFA	Exploratory Factor Analysis
IBM SPSS	IBM Statistical Package for the Social Sciences
KMO	Kaiser–Meyer–Olkin
MS Forms	Microsoft Forms
NESH	National Committee for Research Ethics in the Social Sciences and the Humanities
NVA	Norwegian Research Information Repository
PAF	Principal Axis Factoring
RPD	Recognition-Primed Decision Making
VUCA	Volatile, Uncertain, Complex, and Ambiguous
